# Outcomes of Kidney Perfusion Techniques in Transplantation from Deceased Donors: A Systematic Review and Meta-Analysis

**DOI:** 10.3390/jcm12123871

**Published:** 2023-06-06

**Authors:** Ahmed S. Ghoneima, Richard X. Sousa Da Silva, Martina A. Gosteli, Adam D. Barlow, Philipp Kron

**Affiliations:** 1 Department of HPB and Transplant Surgery, St. James’s University Hospital, Leeds Teaching Hospitals NHS Trust, Leeds LS9 7TF, UK; ahmed.seif.ghoneima@doctors.org.uk (A.S.G.);; 2 Swiss HPB and Transplantation Center, Department of Surgery and Transplantation, University Hospital Zurich, 8091 Zurich, Switzerland; 3 University Library, University of Zurich, 8006 Zurich, Switzerland

**Keywords:** kidney, renal, transplantation, machine, perfusion

## Abstract

The high demand for organs in kidney transplantation and the expansion of the donor pool have led to the widespread implementation of machine perfusion technologies. In this study, we aim to provide an up-to-date systematic review of the developments in this expanding field over the past 10 years, with the aim of answering the question: “which perfusion technique is the most promising technique in kidney transplantation?” A systematic review of the literature related to machine perfusion in kidney transplantation was performed. The primary outcome measure was delayed graft function (DGF), and secondary outcomes included rates of rejection, graft survival, and patient survival rates after 1 year. Based on the available data, a meta-analysis was performed. The results were compared with data from static cold storage, which is still the standard of care in many centers worldwide. A total of 56 studies conducted in humans were included, and 43 studies reported outcomes of hypothermic machine perfusion (HMP), with a DGF rate of 26.4%. A meta-analysis of 16 studies showed significantly lower DGF rates in the HMP group compared to those of static cold storage (SCS). Five studies reported outcomes of hypothermic machine perfusion + O_2_, with an overall DGF rate of 29.7%. Two studies explored normothermic machine perfusion (NMP). These were pilot studies, designed to assess the feasibility of this perfusion approach in the clinical setting. Six studies reported outcomes of normothermic regional perfusion (NRP). The overall incidence of DGF was 71.5%, as it was primarily used in uncontrolled DCD (Maastricht category I-II). Three studies comparing NRP to in situ cold perfusion showed a significantly lower rate of DGF with NRP. The systematic review and meta-analysis provide evidence that dynamic preservation strategies can improve outcomes following kidney transplantation. More recent approaches such as normothermic machine perfusion and hypothermic machine perfusion + O_2_ do show promising results but need further results from the clinical setting. This study shows that the implementation of perfusion strategies could play an important role in safely expanding the donor pool.

## 1. Introduction

Kidney transplantation remains the therapy of choice for patients with end-stage renal disease and is known to improve quality of life and life expectancy [1,2,3]. However, the number of patients waiting for a kidney graft continues to increase and far exceeds the availability of donor grafts. This has pushed transplant centers worldwide to expand their deceased donor pools by accepting grafts from less-ideal donors such as extended criteria donors (ECD), donors after circulatory death (DCD), and, in some countries, even uncontrolled DCD grafts (uDCD, Maastricht I–II) [4]. The results from these organs showed inferior outcomes compared to those of donation after brain death (DBD) [5,6,7]. This is due to the organ damage caused by hypoxia during a period of ischemia at body temperature (also known as warm ischemia time) between the time of asystole and cold perfusion. The effects of warm ischemia time are even more pronounced in uncontrolled DCD (uDCD). The utilization of DCD and marginal organs has created a need for new technologies of organ preservation, assessment, and optimization, moving from a static environment to dynamic perfusion strategies.

The standard method of organ preservation, since the 1970s, has been static cold storage (SCS), where organs are packed and placed on ice in an insulated container using the hypothermic environment to reduce the metabolic rate at a cellular level [8]. However, the need for acceptance of borderline grafts led to the development and introduction of dynamic perfusion strategies, with the aim of assessing, reconditioning, and predicting post-transplant outcomes. There is a variety of approaches to organ perfusion, mainly differing in temperature, the perfusion solution, the use of supplemental oxygen, and the timing of perfusion.

Hypothermic machine perfusion is a technique, developed in 1967 by Belzer, where the kidney is connected to a perfusion circuit in which cooled preservation solution flows through the organ with the aid of a pump [8,9]. This method benefits from the reduction in cellular metabolism, down to 5–10%, triggered by the low temperatures. Furthermore, hemodynamic stimulus is maintained, which improves renal cortical microcirculation during the preservation period [8]. Hypothermic machine perfusion without supplemental oxygen (HMP) has been based on this concept for decades. The most recent development was the addition of oxygen to hypothermic machine perfusion: HMP + O_2_. Oxygen is added to the preservation solution by methods such as membrane oxygenators or simply by inflating oxygen, thereby further reducing the anaerobic pathways within the remaining metabolic activity [10,11,12].

Normothermic machine perfusion (NMP) is also an ex situ perfusion approach that is applied after retrieval to circulate oxygenated perfusate/blood through the organ at temperatures of up to 35 to 38 °C. The setup includes an organ chamber, a perfusion pump, an oxygenator, a heat exchanger, and monitoring devices to measure flow, pressure, and temperature. This technique has the proposed benefit of providing a physiological environment for preservation and the potential of reconditioning the kidney as well as assessing viability and testing function during perfusion [13,14,15].

Normothermic regional perfusion (NRP) is applied in situ in DCD donors. An extra-corporeal membrane oxygenation (ECMO) machine is used to restore circulation with oxygenated blood to the abdominal organs in situ and has the benefit of reducing warm ischemia time and providing a controlled organ-retrieval process whilst protecting the organs from hypoxic injury [16,17,18]. NRP differs from other perfusion techniques because it is deployed within the donor during retrieval. The current standard of care for organ retrieval in DCD donors, against which NRP is being evaluated, is in situ cold perfusion (ICP), in which the cooling of organs is achieved by a single infusion of a cold preservative solution [19].

Due to the fact that more and more borderline grafts are being considered, the use of dynamic perfusion techniques is steadily increasing. The established approaches showed variable degrees of success in improving outcomes, as evidenced by several trials and meta-analyses, especially in organs from high-risk donors [15,17,20,21,22]. They have also introduced additional ways of assessing the viability and function of organs pre-transplant, the potential of optimizing organs by reconditioning, and ways of overcoming logistical challenges by prolonging the preservation period while maintaining better organ function [23,24]. Since a comparison of all clinical available perfusion approaches in kidney transplantation is lacking, this review was conducted to highlight the advantages and disadvantages of the different perfusion techniques.

## 2. Materials and Methods

### 2.1. Study Design

**Figure 1:**
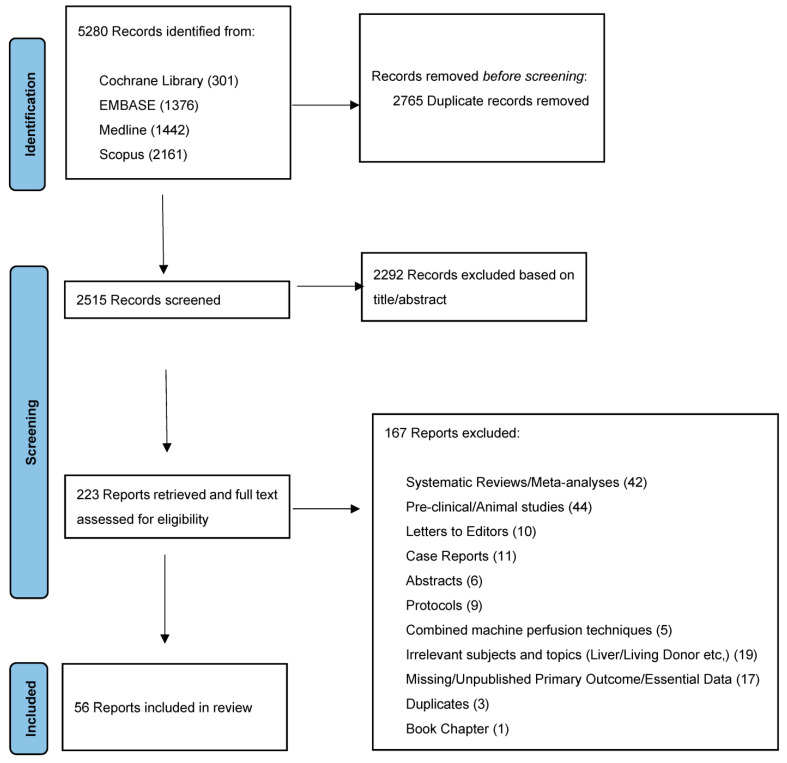
PRISMA.

The study was registered with the PROSPERO database of systematic review protocols (PROSPERO ID: CRD42022379553). The systematic review was conducted according to the Preferred Reporting Items for Systematic Reviews and Meta-Analyses (PRISMA) [25](Figure 1).

Studies between 2010 to 2022 were identified from four electronic databases: MEDLINE (Ovid), Embase (embase.com), Scopus, and the Cochrane Trials Database. The literature search was completed by a professional information specialist from the University Library of the University of Zurich.

### 2.2. Search Strategy

Keywords for the search:
Kidney/renal perfusion
Kidney/renal preservation
Kidney/renal transplantation/transplant/graft/allograft
The specifics of the search strategy are outlined in Supplementary Table S9.
Last search was performed on 30 November 2022.

### 2.3. Outcomes

Primary outcome was the incidence of delayed graft function (DGF), defined as the need for dialysis within 7 days post-transplant.

Secondary outcomes were rates of rejection, graft survival, and patient survival after 1 year.

### 2.4. Data Extraction and Selection

Two reviewers independently reviewed the abstracts. Studies were included based on the following inclusion criteria; human grafts originated from deceased donors, with consecutive transplantation. and the primary outcome was reported.

Studies that fulfilled the following criteria were included in the meta-analyses:

- A comparison between the outcomes (DGF) related to one of the machine perfusion techniques and SCS (i.e., randomized controlled trials, case control trials and observational studies) is included;
- The results in the form of mean and standard deviation are published or can be obtained from median and range [26].

The following types of studies and publications were excluded:

- Secondary studies such as systematic reviews and meta-analyses, as this would lead to duplication in data;
- Pre-clinical studies such as animal and laboratory studies as well as letters to the editor and published protocols;
- Case reports, abstracts, and publications that missed the minimum required data for reporting.

Studies involving combinations of more than one machine perfusion technique in the same patient/organ were excluded, because otherwise the effects of each perfusion approach could not be analyzed separately.

- Suitable studies were identified, and full text analysis was performed. Discrepancies between reviewers were resolved after discussion between them and the senior authors.

### 2.5. Statistical Analysis

Case series and observational studies where the design included outcomes from only one technique of organ perfusion with no comparator were used as descriptive studies to demonstrate rates of incidence and frequencies of the outcomes with different types of machine perfusion in a variety of settings.

Results were presented as percentages for categorical data such as incidence of DGF, as well as graft and patient survival rates at 1 year. Numerical/quantitative data such as ages of donors and recipients, cold ischemia times (CIT), and serum creatinine levels at 1 year were presented as mean and standard deviation (SD) or median and interquartile range (IQR), depending on the mode in the publication.

Included studies were grouped according to technique, as in the aforementioned classification (i.e., HMP, HMP + O_2_, NMP, and NRP), and meta-analyses of the results of each group compared to SCS were performed using RevMan 5.4. A fixed-effect method was used for the analysis of more statistically homogeneous data (I2 < 50); furthermore, a random effects model was run for sensitivity analysis, which did not show any significant difference. For more heterogeneous results (I2 > 50), a random effects model was used to analyze and present data. Mantel–Haenszel approach was used for dichotomous data, and a standard inverse variance approach was used for continuous data. Outcomes were presented in separate forest plots to demonstrate effects and significance using Cochrane Review Manager [27].

## 3. Results

### 3.1. Literature Search and Systematic Review

In total, 5280 records were identified from the literature search based on the keywords, and then 2765 duplicates were removed. Of the 2515 remaining results, 2292 did not fulfill the inclusion criteria based on a title and abstract review. The full texts of 223 reports were assessed for eligibility, and 56 studies were included in the systematic review (Figure 1).

### 3.2. Hypothermic Machine Perfusion (without Oxygen) (HMP)

#### 3.2.1. Descriptive Statistics

A total of 43 studies reported outcomes related to hypothermic machine perfusion (HMP).

The results from 34 studies revealed that 7543 patients received kidneys using HMP, 33.7% of which came from DCD. The mean age of donors was 45.4 years (SD +/−16), and the mean age of recipients was 50.34 years (SD +/−12.97). The overall incidence of DGF in kidneys preserved using HMP was 26.4%. Twenty studies exclusively reported outcomes for DCD kidneys or provided separate outcome results for DCD kidneys. The DGF rate in this subgroup was 45.6%. Ten studies exclusively provided results of ECD kidneys, and the incidence of DGF was 28%.

In nine studies, data were expressed as median with interquartile range (IQR) or 95% CI and, therefore, could not be included in the summary statistics but were maintained in the dataset for completeness.

Supplementary Table S10 contains a summary of the basic information available from all the studies reporting on HMP.

#### 3.2.2. Meta-Analysis

Overall, 22 studies compared HMP to SCS, while 16 studies provided enough information for inclusion in the meta-analysis, representing a total of 7136 patients, 3546 (49.7%) HMP, and 3590 (50.3%) SCS. Supplementary Table S1 outlines the characteristics of the two groups and shows a higher donor age in the HMP group; otherwise, recipient age and the proportion of ECD and CIT were comparable among the two treatment groups (Supplementary Table S1).

**Figure 2:**
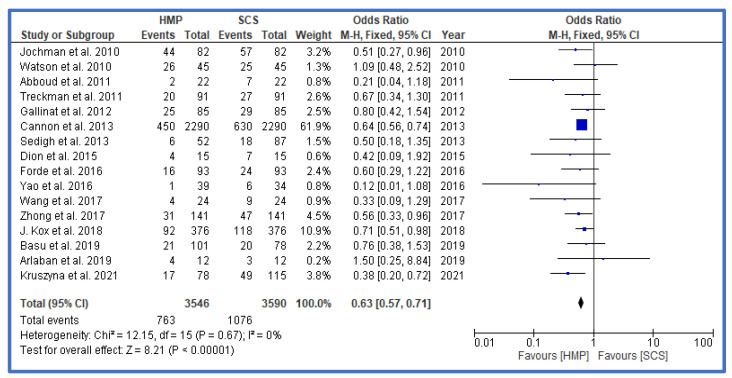
Forest plot—overall rates of DGF in HMP compared to SCS [28,29,30,31,32,33,34,35,36,37,38,39,40,41].

**Figure 3:**
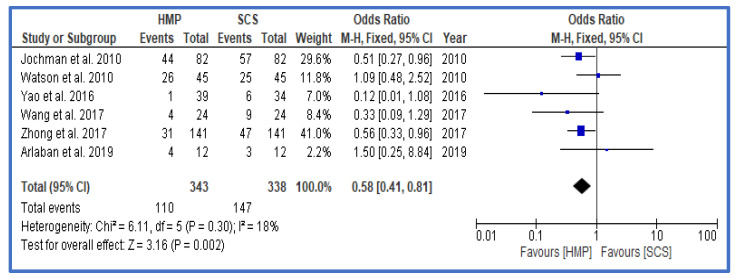
Forest plot—rates of DGF in DCD—HMP compared to SCS [28,32,33,35,40,42].

**Figure 4:**
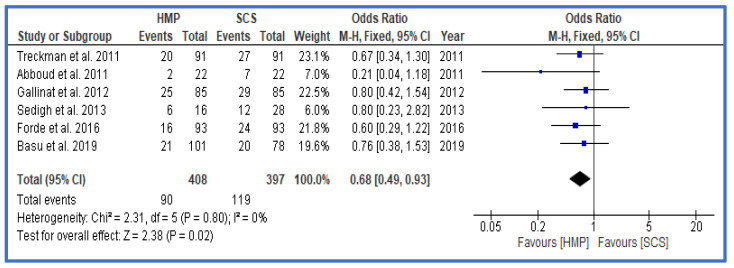
Forest plot—rates of DGF in ECD—HMP compared to SCS [29,30,31,34,37,39].

DGF as the primary outcome was significantly lower in the HMP group vs. the SCS group (Figure 2), with an odds ratio (OR) of 0.63 (0.57, 0.71 confidence interval, CI). Further analysis also showed that, particularly within the group of DCD and ECD grafts, the DGF rates were lower in the HMP group (Figure 3 and Figure 4). A funnel plot to assess possible publication bias showed a fairly symmetrical distribution, indicating a low bias (Supplementary Figure S1). An assessment of the level of evidence and risk for bias for each study is shown in Supplementary Figures S2 and S3 as well as in Supplementary Table S12.

**Figure 5:**
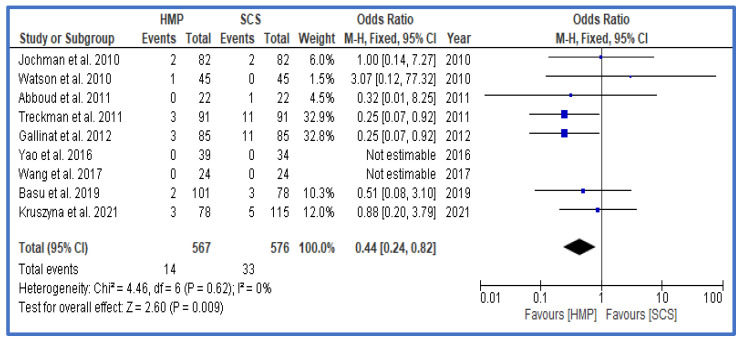
Forest plot—overall rates of PNF in HMP compared to SCS [28,29,30,31,32,33,34,35,41].

The rate of primary non-function (PNF) was also significantly lower in the group treated with HMP in comparison to that of SCS, with an OR of 0.44 (0.24, 0.82) (Figure 5).

**Figure 6:**
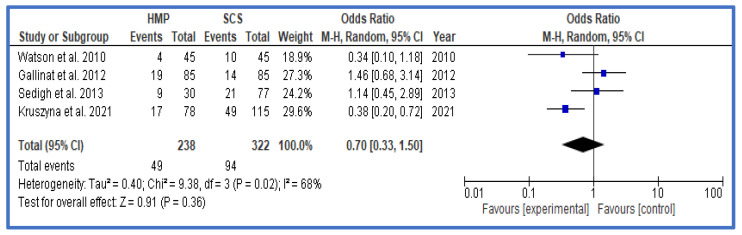
Forest plot—rates of immune rejection at 1 year—HMP compared to SCS [31,35,37,41].

**Figure 7:**
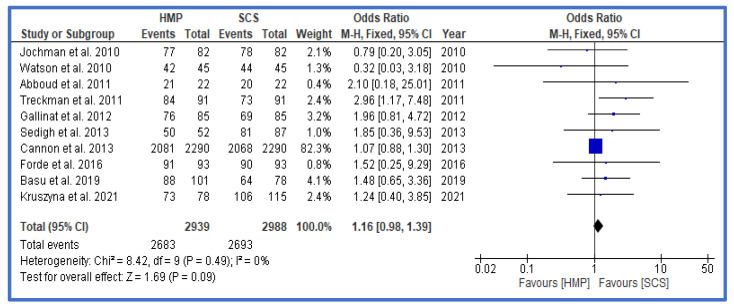
Forest plot—rates of graft survival at 1 year—HMP compared to SCS [28,29,30,31,35,36,37,39,41].

**Figure 8:**
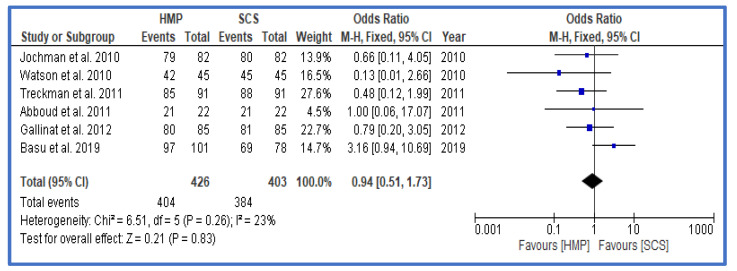
Forest plot—rates of patient survival at 1 year—HMP compared to SCS [28,29,30,31,34,35].

Finally, where available, results of 1-year outcomes were analyzed and presented in the form of rates of rejection (Figure 6), 1-year graft survival (Figure 7), and 1-year patient survival (Figure 8). There was no statistically significant difference for any of these outcomes between HMP and SCS.

### 3.3. Hypothermic Oxygenated Machine Perfusion (HMP + O_2_)

#### 3.3.1. Descriptive Statistics

Five studies assessed the effects of HMP + O_2_, including 263 grafts that were preserved using cold machine perfusion with oxygen. The mean age of donors was 62.5 years (SD +/−6), and the mean age of recipients was 60 years (SD +/−6.5). The overall rate of DGF was 29.7% (Supplementary Tables S2 and S3).

#### 3.3.2. Comparative Studies

Three studies directly compared HMP + O_2_ to SCS. The characteristics in both groups were comparable with a longer CIT in the HMP + O_2_ treated grafts (Supplementary Table S4). All included grafts were from ECD.

**Figure 9:**
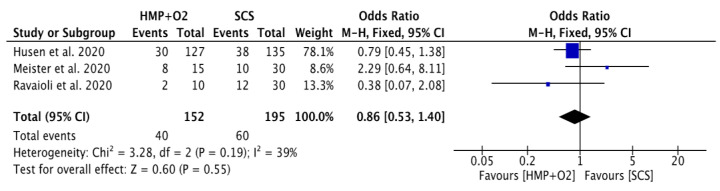
Forest plot—overall rates of DGF in HMP + O_2_ compared to SCS [43,44,45].

Out of a total of 347 grafts, 152 (43.8%) HMP + O_2_ and 195 (56.2%) SCS preserved transplants were included. There was no significant difference between HMP + O_2_ and SCS in rates of DGF, with an OR of 0.86 (0.53, 1.40) for HMP + O_2_ (Figure 9). There was no significant difference between HMP + O_2_ and SCS in the rate of PNF or the 1-year rates of immune rejection, grafts and patient survival.

### 3.4. Normothermic Machine Perfusion (NMP)

#### Descriptive Statistics

Two studies assessing the effects of NMP fulfilled the inclusion criteria. In both studies, NMP was applied end-ischemically after SCS. In both studies, one of the main aims was to assess the safety of this technique and the potential of ex situ viability assessment.

Supplementary Table S5 summarizes the characteristics of the groups that were studied and the findings of both studies.

Due to the limited data that were provided, no further analysis could be performed.

### 3.5. Normothermic Regional Perfusion (NRP)

#### 3.5.1. Descriptive Statistics

Six studies assessing the effects of NRP were within the inclusion criteria. In total, 340 participants received grafts treated with NRP, with a mean age of 42.9 years (SD +/−10.9) for recipients and 47.15 (SD +/−11.5) for donors (Supplementary Table S6). The overall rate of DGF was 71.5% (Supplementary Table S7). This technique is used exclusively in DCD grafts. Overall, 97% of the grafts included in our study were related to uncontrolled/Maastricht I–II DCD (uDCD), which is the highest risk group of donors for organ dysfunction (DGF and PNF) after transplantation.

#### 3.5.2. Comparative Studies

Three studies covered in the systematic review provided enough data to evaluate the effect of NRP compared to that of ICP. In total, 343 grafts were included, of which 80 (23.3%) grafts were treated with NRP and 263 (76.7%) grafts were solely treated with ICP, as is common with the DCD retrieval procedure. There was no significant difference between the ages of the donors and recipients in both groups, and CIT was higher in the ICP group (Supplementary Table S8).

**Figure 10:**
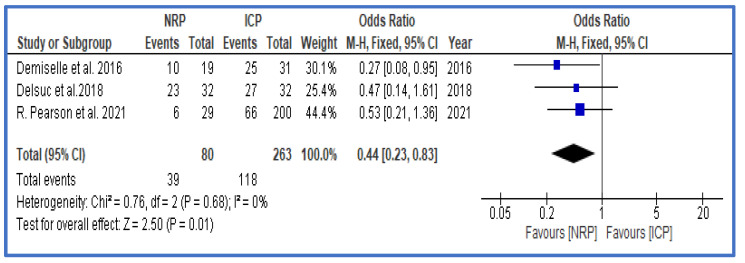
Forest plot—overall rates of DGF in NRP compared to ICP [19,46,47].

A meta-analysis of the results from these studies shows that NRP is associated with significantly lower rates of DGF compared to ICP, with an OR of 0.44 (0.23, 0.83) (Figure 10). The analysis that was conducted did not show any significance between the two groups for PNF, 1-year graft survival, or patient survival.

## 4. Discussion

This systematic review and meta-analysis highlights the importance of different dynamic perfusion techniques, especially new technologies such as HMP + O_2_, and the urgent need to further define their roles in the clinical setting of kidney transplantation. This is particularly relevant in an era of high-risk grafts, given the need to further expand the donor pool. The aim of dynamic preservation strategies is viability testing, reconditioning, and prediction of post-transplant outcomes.

Numerous studies showed worse short- to medium-term outcomes in DCD when compared to DBD, including high DGF rates, despite having comparable results in the long-term [48,49]. These data are supported by a recent meta-analysis that demonstrated higher rates of primary non-function (PNF) (RR 1.43, 1.26–1.62), DGF (RR 2.02, 1.88–2.16) and graft loss after 1 year (RR 1.10, 1.04–1.16) compared to DBD, with similar results for 10-year kidney function and graft loss [50]. Similarly, ECD grafts are known to have higher rates of DGF compared to DBD grafts [51,52].

Based on the findings of the included studies, DGF was used as the primary outcome for this study. There is evidence showing an association between DGF, episodes of rejection, and graft loss. A meta-analysis of 151,594 kidney transplants revealed a 41% increased risk of graft loss (RR 1.41, 1.27–1.56) at 3 years and a 38% relative increased risk of acute rejection (RR 1.38, 1.29–1.47) associated with DGF [53]. Furthermore, DGF has an impact on quality of life with the burden of dialysis sessions and transplant biopsies. DGF is also associated with considerable financial implications. Some studies from North America estimated an USD 18,000–USD 25,000 increase in costs for each episode of DGF due to the additional procedures and investigations [54,55]. Therefore, improvement in DGF rates would translate into improvements in clinical care, patient experience, and service costs. There is evidence showing the clear benefit of machine perfusion approaches in DCD kidney transplantation compared to SCS, but the results of newer machine perfusion approaches, e.g., HMP + O_2_ and normothermic machine perfusion, are limited to preclinical studies, feasibility studies, or studies that could not show any significant advantage of these perfusion strategies [15,20,28,43]. The basic research provided evidence on the mechanism of action and effects on a molecular level, and clinical trials are underway to prove that this is transferrable to practice [11,56,57].

Hypothermic machine perfusion is the most thoroughly studied method of machine perfusion in the literature and the gold standard of preservation for DCD kidneys in many countries worldwide. The review results show that HMP was associated with a significantly lower incidence of DGF compared to SCS (OR 0.63; 0.57, 0.71), despite having a longer CIT. These effects were even more marked in the subgroup analysis of kidneys from DCD, where there was an even lower incidence of DGF with HMP (OR 0.41, 0.81; *p* value < 0.001). The meta-analysis could also show a significantly lower rate of PNF when comparing HMP to SCS. No significant differences could be detected in the 1-year follow-ups comparing HMP to SCS. However, the short-term results justify the use of HMP to improve graft function and reduce the burden caused by DGF, such as additional dialysis sessions, ultrasound scans, and biopsies and a longer hospital stay. This would be even more valuable with marginal grafts at a higher risk of DGF, and there is growing evidence that supports that effect in DCD and ECD [28,29,30,31,32,33,42]. In addition to improving graft function, the clinical applications of HMP can be helpful in overcoming time and logistical challenges. This might help in bridging the lack of operating theater access or patient optimization, with less compromise in the outcomes. Thus, HMP can mitigate the effects of time and distance in the transplantation process, as it was shown to provide good outcomes despite a longer CIT [34,58,59,60,61]. One downside of this perfusion approach is the existing lack of objective parameters that can be assessed during perfusion to predict post-transplant outcomes. A recent Cochrane analysis showed the benefits of HMP compared to SCS, but newer perfusion approaches such as HMP + O_2_ and NMP could not be analyzed [62]. The studies included are heterogeneous regarding the time point when perfusion was started for upfront vs. end-ischemic graft perfusion. There is preclinical evidence showing the advantage of the upfront machine perfusion approach vs. the end-ischemic machine perfusion approach [63], but the damage in the end-ischemic perfusion group is much higher due to the additional cold ischemia time added.

Recent perfusion approaches such as HMP + O_2_ benefit from the same principles of hypothermia and the concepts of dynamic organ preservation such as HMP. Furthermore, the advantage of supplemental oxygen is demonstrated in pre-clinical studies and is already clinically established in liver transplantation [64]. Recently, this knowledge was assessed in the clinical setting of kidney transplantation in two randomized controlled trials (RCT) under the patronage of the COPE consortium. However, the most recent RCT failed to show additional benefits regarding the primary outcome measure, DGF. The study also did not show an advantage of HMP + O_2_ in patient survival or PNF. Looking deeper into the results and assessing the secondary outcomes, the addition of oxygen was able to decrease the number of severe complications (>Clavien Dindo IIIa) to 46 (11%) for 417 HMP + O_2_ (95% CI 8–14%) from 76 (16%) for 474 HMP (95% CI 13–20%) (*p* = 0.032), reduce the biopsy-proven episodes of acute rejections (a relative risk reduction of 44% (relative risk ratio 0.56, 95% CI 0.31–0.98)), and also show lower rates of graft failure with HMP + O_2_ (3 (3%) out of 106) compared with HMP (11 (10%) out of 106, hazard ratio 0.27, and 95% CI 0.07–0.95; *p* = 0.028) [12]. The other multicentric RCT conducted under the same patronage investigated the effects of short-term end-ischemic HMP + O_2_ vs. SCS in ECD grafts. The results of the study could not show any significant differences between the two treatment groups, but this study was probably underpowered. The very high overall graft survival rate contributed to that too [43]. Furthermore, with the limited number of patients included, the underlying graft injury in ECD grafts might be too small to show a significant difference in the included study population.

The results of our systematic review showed that DGF rates in the HMP + O_2_ vs. HMP-treated grafts were comparable (29.7% vs. 26.4%, respectively; ns). However, these results have to be interpreted with caution, as the grafts in the two groups were not comparable and were very heterogeneous. Mainly, all grafts in the HMP + O_2_ treatment group were either ECD or DCD grafts, whereas all donor types were represented in the HMP group. The meta-analysis comparing HMP + O_2_ vs. SCS did not show benefits for DGF, with an OR of 0.86 (0.53, 1.40). Again, the SCS group did not contain any DCD grafts, whereas the main indication for dynamic preservation strategies is grafts from high-risk donor groups. This analysis highlights the lack of clinical evidence assessing the effects of oxygenated perfusion in kidney transplantation. The strong, existing pre-clinical evidence supports intensifying further research to explore the effect of additional oxygen in kidney perfusion in the clinical setting. From a clinical point of view, HMP + O_2_ is easy to apply. Furthermore, HMP + O_2_ has the potential for organ assessment, reconditioning, immune modulation, and a reduction in rejection without many additional financial and time costs [56].

Normothermic machine perfusion (NMP) refers to ex situ perfusion of the organ at near physiological conditions, for organ preservation where the organ is oxygenated, and all metabolic pathways are maintained to keep the cellular functions intact. A deeper investigation of the underlying mechanism showed that normothermic preservation was associated with an increase in proteins mediating the key metabolic processes, including fatty acid ß-oxidation, the tricarboxylic acid cycle, and acid phosphorylation [65]. NMP upregulates certain cellular defense mechanisms, an effect that is similar to ischemic preconditioning, involving HIF proteins as well as pro-inflammatory cytokines such as TNF-a [11].

The effect and benefits of NMP are outlined in a number of pre-clinical studies, including animal models and laboratory experiments, which show the success of this method in preserving organs for prolonged periods of time [66]. This was also deemed successful after human kidneys were transplanted with a good outcome after being preserved at 34 °C using a blood-based solution. The results of our literature search revealed two small clinical studies in which NMP was used to preserve kidneys in a clinical setting for a cohort of patients, in the context of controlled trials that would yield clinically relevant data [67,68]. The rate of DGF in both studies was less for NMP when compared to SCS, but these differences were not significant. Despite the lack of significance, these trials could demonstrate the feasibility and safety of this technique. NMP might offer a solution to logistical issues, such as the lack of theater access, etc. Furthermore, NMP can be used for organ repair and reconditioning up to a certain degree [15]. In addition, this technique might represent a platform for pharmacological, gene, and stem cell therapy, as the intact metabolic pathways permits the administration of these treatments. This is still in the experimental phase for the time being but represents the potential for clinical implementation in the future [69,70]. The downside of this technique is the cost intensity as well as the rather complicated setup before organ perfusion.

Normothermic regional perfusion (NRP) is a technique exclusively used in a DCD setting, which was implemented in 1997 as a method for organ reconditioning before procurement [18,71]. This technique has to be assessed separately from the other techniques (HMP, HMP + O_2_, and NMP), as this perfusion approach is applied before and during retrieval. In our systematic review, six studies fulfilled the inclusion criteria, of which five presented data from uDCD. The inclusion of uDCD grafts might explain the higher DGF rate (71.5%) compared to that of other perfusion approaches. Due to its nature of being an in situ perfusion technique, it was directly compared with in situ cold perfusion (ICP). Here, organs are perfused with cold perfusion fluid in the donor after asystole during organ retrieval. Three studies were identified that compared these two perfusion approaches, including a mixture of DCD and uDCD grafts. The direct comparison of these two in situ perfusion approaches revealed a significant lower rate of DGF, with NRP (OR (95% CI) = 0.44 (0.23, 0.83)). These results have to be interpreted with caution because the number of studies and grafts is limited, and there is a degree of heterogeneity in the study population. In addition, ICP is performed after asystole, resulting in a relevant period of warm ischemia, whereas NRP is started earlier, before organ retrieval is initiated. Other studies showed the benefits of NRP in improving survival, so it is considered a useful method to enlarge the donor pool by allowing the utilization of marginal grafts while maintaining a reasonable safety profile [46]. From a clinical perspective, NRP is established in uDCD/Maastricht I–II, as it was successfully employed by Spanish organ retrieval teams in the “Donor from the street programme” and showed promising results [46,72]. It was recently adopted for DCD/Maastricht III organ donations as well. This is limited by the cost and labor-intensive nature of the technique as well as by the legislation in different countries.

A number of studies highlighted the possibility of using two different machine perfusion techniques in the same graft, combining the benefits of both approaches. Most studies highlighted the combination of NMP and HMP, while a further study suggested controlled oxygenated rewarming, which combines the use of HMP + O_2_ and NMP in a sequential manner [73,74,75,76]. The combination of NMP and HMP could show a higher urinary output, better flow, and higher oxygen consumption compared to NMP alone. Furthermore, preclinical studies combining HMP + O_2_ and NMP could show the benefits of lower renal resistance, better blood flow, and higher oxygen consumption compared to SCS grafts followed by NMP. In line with this, HMP + O_2_ significantly improved the initial perfusion hemodynamics [77]. However, these data are still preliminary and need further clinical validation.

There are a few limitations to our study including heterogeneity, risk of bias, and the effects of missing data.

Although I2 tests of the meta-analyses show reasonable homogeneity, there is a degree of heterogeneity among the study designs. Nevertheless, we felt that this mixture including observational studies and controlled trials allowed for coverage of a wide variety of contexts relevant to machine perfusion in kidney transplantation. Another source of heterogeneity within the data is the different types of donors; however, the inclusion of all types of donors, i.e., DBD, DCD, and ECD, allows for a better representation of all the types of grafts encountered in clinical practice; to overcome this issue, we performed subgroup analyses on the most important types of grafts.

Furthermore, there is a risk of bias as the meta-analysis is affected by the individual studies that make up the dataset, and, since some of the studies included are retrospective studies and not randomized trials, there is the potential for selection bias. Although there is no statistically significant difference between the characteristics of the groups included in the meta-analyses, the risk of selection bias cannot be completely ruled out.

There is also a limitation caused by missing data that are either not published and, therefore, inaccessible or reported in formats that did not allow for integration into the summary statistics and the meta-analyses (e.g., median and interquartile ranges). However, overall, a reasonable amount of information was reported and analyzed to provide the overall conclusion.

## 5. Conclusions

The systematic review and meta-analysis show the benefits of the different perfusion approaches in kidney transplantation. There is evidence directly comparing different perfusion strategies in pre-clinical models, but the transfer into the human setting is still pending. Real-time assessment, graft reconditioning, and the prediction of post-transplant outcomes are hot topics in the transplantation society worldwide, but, so far, objective parameters for the acceptance of grafts or for the prediction of post-transplant outcomes in kidney transplantation are missing. Whereas, the evidence for HMP in kidney transplantation is supported by several studies on newer machine perfusion approaches, such as HMP + O_2_ and NMP in kidney transplantation, which need further evaluation in humans before any clinically relevant conclusions can be drawn.

## Data Availability

Not applicable.

## Supplementary Materials

The following supporting information can be downloaded at: https://www.mdpi.com/article/10.3390/jcm12123871/s1, Supplementary Table S1: Characteristics of HMP and SCS groups included in the meta-analysis; Supplementary Table S2: Study design, donor and recipient characteristics in HMP + O_2_; Supplementary Table S3: HMP + O_2_–outcomes; Supplementary Table S4: Characteristics of HMP + O_2_ and SCS groups included in the meta-analysis; Supplementary Table S5: Characteristics and outcomes of studies related to NMP; Supplementary Table S6: Study design, donor and recipient characteristics in NRP; Supplementary Table S7: NRP—Outcomes; Supplementary Table S8: Characteristics of NRP and ICP groups included in the meta-analysis; Supplementary Table S9: Search Strategy; Supplementary Table S10: Study design, donor and recipient characteristics in HMP; Supplementary Table S11: HMP—Outcomes; Supplementary Table S12: Level of evidence and quality level for bias risk (Newcas-tle-Ottawa score); Supplementary Figure S1: Funnel plot for DGF in studies comparing HMP vs. SCS; Supplementary Figure S2: Risk-of-bias graph and summary table. Authors’ judgments about each risk-of-bias item as percentages among all included HMP vs. SCS studies; Supplementary Figure S3: Risk-of-bias graph and summary table. Authors’ judgments about each risk-of-bias item as percentages among all included HMP + O_2_ vs. SCS studies [12,19,23,28,29,30,31,32,33,34,35,36,37,38,39,40,41,42,43,44,45,46,47,48,58,59,60,61,67,68,72,78,79,80,81,82,83,84,85,86,87,88,89,90,91,92,93,94,95,96,97,98,99,100,101,102].

## Abbreviations

CIT	Cold Ischemia Time
DBD	Donation after Brain Death
DCD	Donation after Circulatory Death
DGF	Delayed Graft Function
ECD	Extended Criteria Donors
ECMO	Extra-Corporeal Membrane Oxygenation
HMP	Hypothermic Machine Perfusion
HOPE	Hypothermic Oxygenated Perfusion
ICP	In situ Cold Perfusion
IQR	Interquartile Range
NMP	Normothermic Machine Perfusion
NRP	Normothermic Regional Perfusion
OR	Odds Ratio
PNF	Primary Non-Function
PRISMA	Preferred Reporting Items for Systematic Reviews and Meta-Analyses
RR	Relative Risk
SCD	Standard Criteria Donors
SCS	Static Cold Storage
SD	Standard Deviation
uDCD	uncontrolled Donation after Cardiac Death
