# Supplementary material for: Outcomes of Kidney Perfusion Techniques in Transplantation from Deceased Donors: A Systematic Review and Meta-Analysis
Source: J Clin Med. 2023 Jun 6;12(12):3871. doi: 10.3390/jcm12123871 (PMC10298857; doi:10.3390/jcm12123871)
Supplement: Supplementary file 1 [file jcm-12-03871-s001.zip › jcm-2311724-Supplementary .docx]

**Supplementary Table S1.** Characteristics of HMP and SCS groups included in the meta-analysis

| **Data point** | **Mean Difference (95% Confidence Interval (CI))** |
| --- | --- |
| Donor age | 1.02 (0.59, 1.45) |
| Recipient age | 0.00 (-0.52, 0.53) |
| ECD | 1.01 (0.89, 1.14) * |
| CIT | 1.71 (-0.35, 3.78) |

* Odds Ratio (95% CI)

**Supplementary Table S2.** Study design, donor and recipient characteristics in HMP+O2

| **Study** | **Study design** | **Number of participants (HMP+O2)** | **Donor age (mean +/-SD years)** | **Type of donor (DBD/ DCD)** | **ECD Status** | **Recipient age**  **(**mean +/-SD **years)** | **Cold Ischaemia Time (CIT)**  **(**mean +/-SD **hours)** |
| --- | --- | --- | --- | --- | --- | --- | --- |
| J. Houtzager et al, 2020 [78] | Case Series | 5 | 43+/-14.5 | DBD + DCD | 0% | 60.6+/-9.4 | 20.1+/-6.8 |
| I. Jochmans et al. 2020 [12] | Randomised Controlled Trial | HMP+O2 106  HMP 106 | HMP+O2  58 (54–63)*  HMP  58 (54–63)* | DCD | - | HMP+O2  60 (53–68)*  HMP  61 (51–65)* | HMP+O2  11·0 (8·7–13·7)  HMP  10·3 (8·9–14) |
| F. Meister et al. 2020 [44] | Controlled Trial ^C^ | HMP+O2 15  SCS 30 | HMP+O2 66+/-12  SCS 66+/-8 | DBD | 100% | HMP+O2 60+/-9  SCS 60+/-10 | HMP+O2 10.7+/-3.8  SCS 11.2 +/- 3.6 |
| M. Ravaioli et al. 2020 [45] | Controlled Trial ^C^ | HMP+O2 10  SCS 30 | HMP+O2  71.5 (60–78)*  SCS  69.5 (59–79)* | DBD | 100% | HMP+O2  61 (50–65)*  SCS  60.5 (48–68)* | HMP+O2  14.5 (10.8–22)*  SCS  14 (8–21)* |
| Husen et al. 2021 [43] | Randomised Controlled Trial ^C^ | HMP+O2 127  SCS 135 | HMP+O2 64 (50-82)*  SCS 65 (51-84)* | DBD | 100% | HMP+O2  63.8 (30.7-81.2)*  SCS  60.9 (22-76.8)* | HMP+O2  13.2 (5.1-28.7)*  SCS  12.9 (4.0-29.2)* |

^- = Not reported HMP = Hypothermic Machine Pefusion^

^N/A = Not applicable^

^Mean+/-SD= mean and Standard Deviation SCS = Static Cold Storage^

^c = Comparative study included in meta-analysis DBD = Donation after Brainstem Death^

^* = Median (range) DCD = Donation after Cardiac Death^

**Supplementary Table S3.** HMP+O2 - outcomes

| **Study** | **DGF** | **DGF DCD** | **DGF ECD** | **PNF** | **Rejection at 1 year** | **1 year graft survival** | **1 year patient survival** | **Conclusion** |
| --- | --- | --- | --- | --- | --- | --- | --- | --- |
| J. Houtzager et al, 2020 [78] | 30% | N/A | N/A | 10% | - | - | - | HMP+O2 is feasible and safe |
| I. Jochmans et al. 2020 [12] | HMP+O2 36%  HMP 36% | HMP+O2 36%  HMP 36% | - | HMP+O2 3%  HMP 5% | HMP+O2 14%  HMP 26% | HMP+O2 97%  HMP 90% | HMP+O2 93%  HMP 93% | HMP+O2 is associated with improved function and reduces rejection in DCD |
| F. Meister et al. 2020 [44] | HMP+O2  53%  SCS  33% | N/A | HMP+O2  53%  SCS  33% | HMP+O2 7%  SCS  0% | - | - | - | Renal resistance in HMP+O2 |
| M. Ravaioli et al. 2020 [45] | HMP+O2  20%  SCS  40% | N/A | HMP+O2  20%  SCS  40% | HMP+O2  0%  SCS3.3% | - | HMP+O2 100%  HMP 93.3% | HMP+O2 100%  HMP 96.6% | HMP+O2 is associated with lower rates of DGF |
| Husen et al. 2021 [43] | HMP+O2  23.6%  SCS  28.1% | N/A | HMP+O2  23.6%  SCS  28.1% | HMP+O2  6.3%  SCS  5.9% | HMP+O2  18.1%  SCS  13.3% | HMP+O2  92.1%  SCS  93.3% | HMP+O292.9%  SCS  9 | No difference between HMP+O2 and SCS in ECD DBD |

PNF = Primary Non-function N/A = Not applicable

DGF = Delayed Graft Function HMP = Hypothermic Machine Perfusion

DCD = Donation after Cardiac Death SCS = Static Cold Storage

ECD = Extended Criteria Donor HMP+O2 = Hypothermic Oxygenated Machine Perfusion

DBD = Donation after Brainstem Death - = Not reported

**Supplementary Table S4.** Characteristics of HMP+O2 and SCS groups included in the meta-analysis

| **Data point** | **Mean Difference (95% Confidence Interval (CI))** |
| --- | --- |
| Donor age | -0.96 (-2.17, 0.25) |
| Recipient age | 1.99 (-1.69, 5.67) |
| CIT | 0.95 (0.19, 1.71) |

**Supplementary Table S5.** Characteristics and outcomes of studies related to NMP

| **Study** | **Number of participants (NMP)** | **Donor age**  **Median (IQR)** | **Recipient age**  **Median (IQR)** | **Type of donor** | **Cold ischaemia Time**  **Mean +/- SD** | **DGF** | **PNF** |
| --- | --- | --- | --- | --- | --- | --- | --- |
| M. Nicholson et al. 2013 [67] | NMP 18  SCS 47 | NMP 61+/-1  SCS 62+/-6 | NMP 58+/-12  SCS 56+/-10 | DBD + DCD | NMP  10.6hr+/-39min and 26+/-20min  SCS 11.8+/-3.8 | NMP  5.6%  SCS  36.2% | NMP 0%  SCS  2% |
| P. Chandak et al. 2019 [68] | NMP 7  SCS 7 | 65 (48–69)* | NMP  48 (29–61)*  SCS  57 (31–61)* | DBD + DCD | NMP 13.97+/-1.75  SCS 10.13+/-2.783 | NMP  14%  SCS  43% | NMP  0%  SCS  20%S |

NMP = Normothermic Machine Pefusion DGF = Delayed Graft Function

N/A = Not applicable PNF = Primary Non-function

Mean+/-SD= mean and Standard Deviation SCS = Static Cold Storage

DBD = Donation after Brainstem Death Median (IQR) = Median and Interquartile Range

DCD = Donation after Cardiac Death

**Supplementary Table S6.** Study design, donor and recipient characteristics in NRP

| **Study** | **Study design** | **Number of participants (NRP)** | **Donor age (mean +/-SD years)** | **Type of donor** | **Recipient age**  **(mean +/-SD years)** | **Cold Ischaemia Time (CIT)**  **(mean +/-SD hours)** |
| --- | --- | --- | --- | --- | --- | --- |
| O. Reznik et al. 2010 [72] | Case Series | 8 | 66.5 (45 – 78)* | uDCD | 53.4 (36-67)* | - |
| O. Reznik et al. 2010 [79] | Case Series | 16 | 44.1 (22-55)* | uDCD | 53.5 (36-67)* | - |
| N. Miranda-Utrera et al. 2015 [80] | Observational Study | NRP/uDCD 236  SCS/DBD 260 | NRP 42.04 ± 10.7  SCS 40.92 ± 10.9 | uDCD  DBD | NRP 47.15 ± 11.3  SCS 45.22 ± 11.4 | NRP 12.9 ± 5.8  SCS 18.4 ± 4.6 |
| J. Demiselle et al. 2016 [46] | Observational Study ^C^ | NRP 19  ICP 31 | NRP 45.7 +/- 5.7  ICP 45.3 +/- 7.2 | uDCD | NRP 41.4 +/- 10.1  ICP 43.8 +/- 10.2 | NRP 11.2+/-3.57  ICP 12.6+/-3.1 |
| C. Delsuc et al.2018 [19] | Observational Study ^C^ | NRP 32  ICP 32 | NRP 43.2 ± 8.6  ICP 41.8 ± 10.1 | uDCD | NRP 47.9 ± 10.7  ICP 45.8 ± 11.1 | NRP 13.6+/-3.5  ICP 17.1+/- 4.167 |
| R. Pearson et al. 2021 [47] | Observational Study ^C^ | NRP 29  ICP 200 | NRP 42(19-70)*  ICP 54 (2-79)* | DCD | NRP 49.4 (11–73)*  ICP 54.4 (23–83)* | NRP 9.3 (4.3–16.85)*  ICP 11.1 (6–24)* |

- = Not reported NRP = Normothermic Regional Perfusion

N/A = Not applicable SCS = Static Cold Storage

c = Comparative study included in meta-analysis DBD = Donation after Brainstem Death

* = Median (range) DCD = Donation after Cardiac Death

ICP = In situ Cold Perfusion uDCD = uncontrolled Donation after Cardiac Death

**Supplementary Table S7.** NRP – Outcomes

| Study | DGF | PNF | 1 year graft survival | 1 year patient survival | Conclusion |
| --- | --- | --- | --- | --- | --- |
| O. Reznik et al. 2010 [72] | 37.5% | 0% | - | - | NRP used for resuscitating DCD kidneys with ischemic damage in uncontrolled DCD |
| O. Reznik et al. 2010 [79] | 62.5% | 0% | - | - | NRP used for resuscitating DCD kidneys with ischemic damage in uncontrolled DCD |
| N. Miranda-Utrera et al. 2011 [80] | NRP 80.9%  SCS 46.8% | NRP 5.5%  SCS 4% | NRP 92.8%  SCS 93.6% | NRP 99.1%  SCS 98.6% | High rates of DGF with uDCD despite NRP but good 1 year results |
| J. Demiselle et al. 2016 [46] | NRP 53%  ICP 81% | NRP 5.2%  ICP 6.7% | - | - | NRP improves rates of DGF in uDCD |
| C. Delsuc et al.2018 [19] | NRP 72%  ICP 84% | NRP 3%  ICP 3% | NRP 96.5%  ICP 96.8% | NRP 92.6%  ICP 100% | NRP improved 1 year graft function but not significantly altered short term outcomes |
| R. Pearson et al. 2021 [47] | NRP 20.7%  ICP 35% | - | NRP 96.6%  ICP 92.7% | NRP 92.6%  ICP 100% | NRP improves rates of DGF in DCD |

PNF = Primary Non-function NRP = Normothermic Regional Perfusion

DGF = Delayed Graft Function SCS = Static Cold Storage

DCD = Donation after Cardiac Death N/A = Not applicable

DBD = Donation after Brainstem Death - = Not reported

uDCD = Uncontrolled DCD ICP = In situ Cold Perfusion

**Supplementary Table S8.** Characteristics of NRP and ICP groups included in the meta-analysis

| **Data point** | **Mean Difference (95% Confidence Interval (CI))** |
| --- | --- |
| Donor age | -0.39 (-2.86, 2.07) |
| Recipient age | -2.49 (-5.74, 0.77) |
| CIT | -2.83 (-3.73, -1.93) |

**Literature Search for the Systematic Review**

**Supplementary Table S9**. Search Strategy

| MEDLINE (Ovid MEDLINE(R) and Epub Ahead of Print, In-Process, In-Data-Review & Other Non-Indexed Citations and Daily) |
| --- |
| #1 ((kidney* or renal*) adj3 (perfus* or reperfus* or hypoperfus* or microperfus* or preserv*)).ti,ab.  #2 exp Kidney Transplantation/ or ((kidney* or renal*) adj3 (transplant* or graft* or allograft*)).ti,ab.  #3 1 and 2  #4 limit 3 to yr="2010 -Current"  #5 limit 4 to (english or german) |
| Embase |
| #1 'kidney perfusion'/exp OR (((kidney* OR renal*) NEAR/3 (perfus* OR reperfus* OR hypoperfus* OR microperfus*)):ti,ab) OR 'kidney preservation'/exp OR (((kidney* OR renal*) NEAR/3 preserv*):ti,ab)  #2 'kidney transplantation'/exp OR (((kidney* OR renal*) NEAR/3 (transplant* OR graft* OR allograft*)):ti,ab)  #3 #1 AND #2  #4 #1 AND #2 AND [2010-2022]/py  #5 #1 AND #2 AND [2010-2022]/py AND [conference abstract]/lim  #6 #1 AND #2 AND [2010-2022]/py NOT [conference abstract]/lim  #7 #1 AND #2 AND [2010-2022]/py NOT [conference abstract]/lim NOT ([animals]/lim NOT [humans]/lim)  #8 #1 AND #2 AND [2010-2022]/py NOT [conference abstract]/lim NOT ([animals]/lim NOT [humans]/lim) AND ([english]/lim OR [german]/lim) |
| Scopus |
| TITLE-ABS-KEY ( ( kidney* OR renal* ) W/3 ( perfus* OR reperfus* OR hypoperfus* OR microperfus* ) ) OR TITLE-ABS-KEY ( ( kidney* OR renal* ) W/3 ( preserv* ) ) ) AND ( TITLE-ABS-KEY ( ( kidney* OR renal* ) W/3 ( transplant* OR graft* OR allograft* ) ) ) AND ( LIMIT-TO ( PUBYEAR , 2022 ) OR LIMITTO ( PUBYEAR , 2021 ) OR LIMIT-TO ( PUBYEAR , 2020 ) OR LIMIT-TO ( PUBYEAR , 2019 ) OR LIMIT-TO ( PUBYEAR , 2018 ) OR LIMIT-TO ( PUBYEAR , 2017 ) OR LIMITTO ( PUBYEAR , 2016 ) OR LIMIT-TO ( PUBYEAR , 2015 ) OR LIMIT-TO ( PUBYEAR , 2014 ) OR LIMIT-TO ( PUBYEAR , 2013 ) OR LIMIT-TO ( PUBYEAR , 2012 ) OR LIMITTO ( PUBYEAR , 2011 ) OR LIMIT-TO ( PUBYEAR , 2010 ) ) AND ( LIMITTO ( LANGUAGE , "English" ) OR LIMIT-TO ( LANGUAGE , "German" ) ) |
| Cochrane Trials Database |
| #1 ((kidney* or renal*) NEAR/3 (perfus* OR reperfus* OR hypoperfus* OR microperfus*)):ti,ab,kw OR ((kidney* or renal*) NEAR/3 (preserv*)):ti,ab,kw  #2 ((kidney* or renal*) NEAR/3 (transplant* OR graft* OR allograft*)):ti,ab,kw  #3 #1 AND #2  #4 #1 AND #2 with Publication Year from 2010 to present, in Trials |

**Results of the systematic review of publications related to Hypothermic**

**Machine Perfusion without supplemental Oxygen.**

**Supplementary Table S10** Study design, donor and recipient characteristics in HMP

| Study | Study design | Number of participants (HMP) | Donor age (mean +/-SD years) | Type of donor (DCD%) | ECD Status | Recipient age  (mean +/-SD years) | Cold Ischaemia Time (CIT)  (mean +/-SD hours) |
| --- | --- | --- | --- | --- | --- | --- | --- |
| F. Cantena et al. 2010 [81] | Case Series | 10 | 63.1+/-6.3 | DBD | 100% | 64.3+/-5.2 | 18.3+/-1.3 |
| G. Ciancio et al. 2010 [61] | Observational Study | 339 | 35+/-15.1 | DBD +  DCD (39%) | 10.9% | 50.2+/-13.5 | 33.2+/-8.4 |
| J. Guarrera et al. 2020 [82] | Case Series | 11 | 45.8+/-13 | DBD | - | 48.6+/-11 | 26.5+/-9.8 |
| I. Jochman et al. 2010 [28] | Randomised Controlled Trial ^C^ | 82 | 43 (17–67)* | DCD | - | HMP  49 (24–73)*    SCS  52 (24–77)* | HMP  15.0 (4.3–28.9)*  SCS  15.9 (8.6–46.6)* |
| N. Matsuno et al. 2010 [83] | Observational Study | 17 | 43.9+/-12.4 | uDCD | - | 41.8+/-10.9 | 8.7+/-6.4 |
| C. Moers et al. 2010 [84] | Case Series | 306 | 50 (16–78)* | DBD +  DCD (24.5%) | 28% | 53 (11–79)* | 15 (4–30)* |
| C. Watson et al. 2010 [35] | Randomised Controlled Trial ^C^ | HMP 45  SCS 45 | 45.6+/-14.6 | DCD | - | HMP  50.3+/-14.2  SCS  48.6+/-13.9 | HMP  13.9 h (6.7-24.2)*  SCS  14.3 h (7.0- 30.1)* |
| I. Abboud et al. 2011 [29] | Controlled Trial ^C^ | HMP 22  SCS 22 | 57.8+/-15.1 | DBD | 100% | HMP  56.5+/-11.0  SCS  53.0+/-15.4 | HMP  20.3+/-8.1  SCS  22.2+/-10.2 |
| De Vries et al. 2011 [103] | Case Series | 430 | 45+/-16 | DCD + uDCD | - | 50+/-14 | 28+/-8 |
| I. Jochmans 2011 [23] | Case Series | 335 | 51 (16–81)* | DBD +  DCD (12.5%) | 27.1% | 53 (11–79)* | 15 (3–30) |
| J. Treckmann et al. 2011 [30] | Randomised Controlled Trial ^C^ | HMP 91  SCS 91 | 66 (50–81)* | DBD | 100% | HMP  65 (20–79)*  SCS  65 (32–79)* | HMP  13 (3–23)*  SCS  13 (4–29)* |
| G. Ciancio et al. 2012 [85] | Observational Study | 132 | 36.9+/-13.9 | DBD +  DCD (1.5%) | - | 51.55+/-12.76 | 31.8+/-9 |
| A. Gallinat et al. 2012 [31] | Randomised Controlled Trial ^C^ | HMP 85  SCS 85 | 70 (65–83)* | DBD | 100% | HMP  66 (39–79)*  CSCS  66 (37–79)* | HMP  11 (4–24)*  SCS  10.5 (3–24)* |
| S. Patel et al. 2012 [86] | Case Series | 73 | 47+/-15 | DBD +  DCD (4.1%) | 27.4% | - | 18.2+/-5.6 |
| R. Cannon et al. 2013 [36] | Observational Study ^C^ | HMP 2290  SCS 2290 | 41.3+/-15.1 | DBD +  DCD (7.7%) | 20% | HMP  53.5+/-13.3  SCS  53.4+/-12.5 | HMP  23.4+/-11.0  SCS  17.4+/-8.4 |
| P. Hoogland et al. 2013 [87] | Case Series | 335 | 45+/-16 | DCD + uDCD | - | 51+/-13 | 27+/-6 |
| M. Nagelschmidt et al. 2013 [88] | Case Series | 111 | 64.1+/-6.6 | DBD + DCD | - | - | 13.8+/-5.3 |
| A. Sedigh et al. 2013 [37] | Controlled Trial ^C^ | HMP 52  SCS 87 | HMP  59 (15–78)*  SCS  59 (2–83) | DBD | HMP 69.2%  SCS  67.8% | HMP  61 (22–75)*  SCS  58 (24–79) | HMP 12.8 (7.0–24.5)*  SCS 11.7 (5.3–25.0) |
| M. Wszola et al. 2013 [89] | Randomised Controlled Trial | 50 | - | DBD | 48% | 50+/-15.1 | 28+/-8.4 |
| I. Hall et al. 2014 [90] | Case Series | 428 | 54+/-15.4 | DBD +  DCD (33%) | 31% | 46.8+/-14.0 | 18.37+/-6.08 |
| M. Dion et al. 2015 [38] | Controlled Trial ^C^ | HMP 15  SCS 15 | 37.4+/-15.2 | DBD +  DCD (53%) | 20% | HMP  45.6±9.3  SCS  51.1±15.2 | HMP  18.783±6.83  SCS  8.767±6.4167 |
| A. Guy et al. 2015 [60] | Controlled Trial | HMP 74  SCS 101 | HMP 52 (40 to 60)**  SCS 51 (35 to 61)** | DBD +  DCD (12%) | HMP 36%  SCS 35% | HMP  51 (44 - 57)**  SCS  50 (39 - 57)** | HMP 23.85 (19.30 -26.62)**  SCS 13.00 (11.79 - 15.36)** |
| A. Guy et al. 2015 [91] | Case Series | 26 | 47 (40-60)** | DBD + DCD | - | 50 (44-54)** | 23 (16-27)** |
| J. Forde et al. 2016 [39] | Observational Study ^C^ | HMP 93  SCS 93 | HMP 58.3 (51–73)*  SCS 55.7  (50–64)* | DBD | 100% | HMP  59.2 (38–75)*  SCS  59.9 (40 to 75)* | HMP 15.6 (7–32)*  SCS 17.9 (9–28)* |
| S. Paloyo et al. 2016 [92] | Observational Study | 236 | 35.95+/-14.4461 | DBD +  DCD (25%) | - | 50.625+/-12.72 | 35.9+/-9.49 |
| C. Parikh et al. 2016 [93] | Observational Study | 671 | 46.8+/-13.7 | DBD +  DCD (26%) | 31% | 54.7+/-15 | 18 (14–22)** |
| L. Yao et al. 2016 [32] | Controlled Trail ^C^ | HMP 39    SCS 34 | 47 (19–57)* | DCD | - | HMP  40 (19-66)*  SCS  47 (20-59)* | - |
| W. Wang et al. 2017 [33] | Randomised Controlled Trial ^C^ | HMP 24  SCS 24 | 48.2+/-12.6 | DCD | 16.7% | HMP  44.6+/-8.1  SCS  40.2+/-6.2 | - |
| Z. Zhong et al. 2017 [42] | Randomised Controlled Trial ^C^ | HMP 141  SCS 141 | 32.8+/-12.4 | DCD | - | HMP  41.4+/-11.6  SCS  40.6 +/- 9.3 | HMP 10.3 (5.1–24.0)*  SCS 11.8 (6.3–22.5)* |
| G. Chen et al. 2018 [94] | Case Series | 58 | 53.1+/- 4.8 | DCD | 100% | 38.1+/-11.7 | 6.7+/-3.5 |
| C. Ding et al. 2018 [95] | Case Series | 76 | 45.3+/-10.7 | DCD + uDCD | - | 39.6+/-7 | 9.2+/-2.2 |
| J. Kox et al. 2018 [96] | Randomised Controlled Trial ^C^ | HMP  376  SCS  376 | 49.6 (16-81)* | DBD +  DCD (21.8%) | 27.9% | HMP  53 (11-79)*  SCS  52 (2-79)* | HMP  15.15 (3-47)*  SCS  15.7 (3-30)* |
| C. Matos et al. 2018 [59] | Controlled Trail | HMP  54  SCS  101 | HMP  42.5 (27.25-50.75)**  SCS  43 (29-52)** | DBD | HMP  18.5%  SCS  28.7% | HMP  44.5 (37.25- 54.75)**  SCS  55 (45- 60)** | HMP  22 (20-23)**  SCS  31.5 (28-34)** |
| K. Patel et al. 2018 [97] | Observational Study | HMP  3665  SCS  864 | HMP  54 (41- 63)**  SCS  54 (43- 63)** | DBD + DCD | - | HMP  56 (47- 64)**  SCS  55 (46- 63)** | HMP  14.8 (11.4- 18.8)**  SCS  14.1 (11.1- 17.2)** |
| M. Arlaban et al. 2019 [40] | Controlled Trail ^C^ | HMP  12  CS  12 | 51.4+/- 11.9 | DCD | 25% | HMP  51+/-10.1  SCS  52.6+/-14.8 | HMP  20.06+/-6.18  SCS  6.08+-1.355 |
| A. Basu et al. 2019 [34] | Observational Study ^C^ | HMP 101  SCS 78 | HMP 62.2+/-6.9  SCS 60.6+/-6.1 | DBD | 100% | HMP  60.7+/-9.9  SCS  62.2+/-9.1 | HMP  28.9+/-10.0  SCS  24.0+/-7.9 |
| L. Samoylova et al. 2019 [98] | Observational Study | HMP 46017  SCS 33283 | HMP 45 (30-55)**  SCS 38 (23-49)** | DBD + DCD | - | HMP  54 (44-63)**  SCS  46 (46-64)** | HMP  19.5 (14.4-25.9)**  SCS  14.5 (10.0-20.0)** |
| M. Sevinc et al. 2019 [99] | Case Series | 128 | 51 (39-63.75)** | DCD | - | 55.5 (47-63)** | 20.1+/-4.87 |
| M. Wszola et al. 2019 [100] | Case Series | 379 | 45.9 +/-15 | DCD | 31.4% | 48+/- 2 | 28.8+/- 8 |
| T. De Sandes Freitas et al. 2020 [58] | Observational Study | HMP 103  SCS 103 | 30 (21–40)*** | DBD | 4.9% | HMP  39.9+/-18.2  SCS  45.9+/-15.0 | HMP  15.8+/-6.2  SCS  20.8+/-4.1 |
| V. Krishnamoorthye et al. 2020 [101] | Observational Study | HMP 729  SCS 932 | HMP 43(28-53)** | DBD + DCD | 9.7% | HMP  57 (46-66)**  SCS  52 (39-62)** | HMP  17 (12.3-21.9)**  SCS  15.8 (11.0, 22.1)** |
| T. Kruszyna et al. 2021 [41] | Observational Study ^C^ | HMP 78  SCS 115 | HMP  44.5 (15-67)*  SCS  44 (5-72)* | DBD | HMP  25.6%  SCS  22.6% | HMP  50 (20-69)*  SCS  49 (20-77)* | HMP  23.2 (6.5-34)*  SCS  20 (5.5-33)* |
| N. Singh et al. 2021 [102] | Observational Study | HMP 39  SCS 581 | HMP 36 (25-48)**  SCS 31 (22-42)** | DBD + DCD | - | - | - |

^- = Not reported HMP = Hypothermic Machine Perfusion^

^N/A = Not applicable SCS = Static Cold Storage^

^c = Comparative study included in meta-analysis DBD = Donation after Brainstem Death^

^* = Median (range) DCD = Donation after Cardiac Death^

^** = Median (Interquartile range) uDCD = uncontrolled Donation after Cardiac Death^

^***= Mean (95% Confidence Interval) Mean+/-SD= mean and Standard Deviation^

**Supplementary Table S11.** HMP - Outcomes

| Study | DGF | DGF DCD | DGF ECD | PNF | Rejection at 1 year | 1 year graft survival | 1 year patient survival | Conclusion |
| --- | --- | --- | --- | --- | --- | --- | --- | --- |
| F. Cantena et al. 2010 [81] | 10% | N/A | 10% | 0% | 20% | 90% | 100% | Celsior solution safe for HMP |
| G. Ciancio et al. 2010 [61] | 4.4% | 25% | - | 1.5% | 9.1% | 94% | - | Long HMP times (>24 hours) have favourable outcomes |
| J. Guarrera et al. 2010 [82] | 36% | N/A | - | - | 27% | 81% | 100% | Poor perfusion parameters predicts DGF but not long term outcomes |
| I. Jochman et al. 2010 [28] | HMP 53.7%  SCS  69.5% | HMP 53.7%  SCS  69.5% | - | HMP 2.4%  SCS 2.4% | - | HMP 93.9%  SCS  95.1% | HMP  96.3%  SCS  97.6% | HMP reduced the risk of DGF in DCD |
| N. Matsuno et al. 2010 [83] | 95% | 95% | - | 0% | - | - | - | Use of Perfusion Pressure to assess viability but not DGF in uDCD |
| C. Moers et al. 2010 [84] | 25% | 53% | - | 2.3 | - | 95% | - | GST, NAG, and H-FABP Predicted DGF, Lactate dehydrogenase, aspartate aminotransferase, and alanine-aminopeptidase NO predictive value |
| C. Watson et al. 2010 [35] | HMP  57.8%  SCS  55.6% | HMP  57.8%  SCS  55.6% | - | HMP2.2%  SCS0% | HMP  9%  SCS  23% | HMP  93.3%  SCS  98% | HMP  93%  SCS  100% | No significant improvement of DGF with HMP in DCD |
| I. Abboud et al. 2011 [29] | HMP  9%  SCS  31% | N/A | 9% | HMP  0%  SCS  4.5% | - | HMP  95%  SCS  91% | HMP  95%  SCS  95% | HMP reduces rates of DGF in ECD |
| De Vries et al. 2011 [103] | 61% | 61% | - | 19.5% | - | 74% | 76% | High renovascular resistance is an independent risk factor of PNF in DCD |
| I. Jochman 2011 [23] | 19% | 50% | 22% | 2% | - | 92% | 97% | Renovascular resistance is an independent risk factor for DGF and 1 year graft loss |
| J. Treckmann et al. 2011 [30] | HMP  22%  SCS  29.7% | N/A | HMP  22%  SCS29.7% | HMP  3%  SCS12% | HMP  18.7%  SCS  14.3% | HMP  92.3%  SCS  80.2% | HMP  93.4%  SCS  96.7% | HMP Reduces DGF and improves 1 year graft survival in ECD DBD |
| G. Ciancio et al. 2012 [85] | 3% | 100% | - | 0.7% | 10.5% | 88.6% | 90.1% | Longer HMP times do not affect graft outcomes |
| A. Gallinat et al. 2012 [31] | HMP 29.4%  SCS 34.1% | N/A | HMP 29.4%  SCS 34.1% | HMP 3.5%  SCS 12.9% | HMP  22.4%  SCS  16.5% | HMP  89%  SCS  81% | HMP  94.2%  SCS  95% | Lower rates of PNF and better 1 year graft survival with HMP in ECD |
| S. Patel et al. 2012 [86] | 52% | - | - | 0% | - | 89% | - | High renovascular resistance is associated with DGF |
| R. Cannon et al. 2013 [36] | HMP 19.7%  SCS  27.5% | - | - | - | - | HMP  90.9%  SCS  90.3% | - | HMP reduces rates of DGF |
| P. Hoogland et al. 2013 [87] | 61% | 61% | - | 20% | - | - | - | LDH and IL-18 associated with PNF in the multivariate  Analyses in DCD |
| M. Nagelschmidt et al. 2013 [88] | 25.2% | - | - | 1.8% | - | - | - | Lipid peroxidation markers (tGST, a-GST, and LPOP) can be used as predictors of DGF |
| A. Sedigh et al. 2013 [37] | HMP 11.5%  SCS  20.7% | N/A | HMP  16.7%  SCS  20.3% | - | HMP 30%  SCS 27.3% | HMP 96%  SCS  93% | - | HMP reduces DGF in DBD |
| M. Wszola et al. 2013 [89] | 32% | N/A | - | - | - | 88% | - | Better 1 year graft survival rate with Pulsatile flow |
| I. Hall et al. 2014 [90] | 32% | 44% | 28% | - | - | - | - | Pi-GST is associated with DGF |
| M. Dion et al. 2015 [38] | HMP 27%  SCS  47% | - | - | 0% | - | - | - | HMP reduces RI |
| A. Guy et al. 2015 [60] | HMP  27%  SCS  47% | HMP 56%  SCS  65% | - | HMP 0%  SCS  2% | - | - | - | HMP is associated with lower DGF rates despite longer CIT |
| A. Guy et al. 2015 [91] | 27% | - | - | 0% | - | - | - | Metabolomics (glucose, inosine, leucine, gluconate) are associated with DGF |
| J. Forde et al. 2016 [39] | HMP 17.2 %  SCS 25.8 % | N/A | HMP 17.2 %  SCS 25.8 % | - | - | HMP 97.85%  SCS  96.77% | - | HMP is associated with lower rates of DGF in ECD |
| S. Paloyo et al. 2016 [92] | 13.1% | 30.5% | - | 0% | - | 92% | - | Longer CIT before HMP is associated with higher DGF |
| C. Parikh et al. 2016 [93] | 34% | - | - | 2% | - | - | - | Biomarkers and pump parameters were associated with DGF |
| L. Yao et al. 2016 [32] | HMP 2.6%  SCS 17.6% | HMP 2.6%  SCS 17.6% | - | 0% | - | - | - | HMP is associated with lower rates of DGF in DCD |
| W. Wang et al. 2017 [33] | HMP 16.7%  SCS 37.5% | HMP 16.7%  SCS 37.5% | - | 0% | - | - | - | HMP is associated with lower rates of DGF in DCD |
| Z. Zhong et al. 2017 [42] | HMP  22.0%  SCS  33.3% | HMP  22.0%  SCS  33.3% | - | - | - | HMP  98%  SCS  93% | - | HMP is associated with lower rates of DGF in DCD |
| G. Chen et al. 2018 [94] | 44% | 44% | 44% | 5.1% | - | - | - | Resistive Indexes can predict DGF in ECD DCD |
| C. Ding et al. 2018 [95] | 26.3% | 26.3% | - | - | - | 88.1% | 92.1% | Increasing MP pressure does not affect DGF |
| J. Kox et al. 2018 [96] | HMP  24.5%  SCS  31.4% | - | - | - | - | - | - | HMP is associated with lower DGF rates |
| C. Matos et al. 2018 [59] | HMP 61.1%  SCS  79.2% | N/A | - | - | - | - | - | HMP improves rates of DGF with long CIT |
| K. Patel et al. 2018 [97] | HMP  34.2%  SCS  42% | - | - | - | - | - | - | HMP reduces rates of DGF |
| M. Arlaban et al. 2019 [40] | HMP 33.3%  SCS  25% | HMP 33.3%  SCS  25% | - | - | - | - | - | No difference between HMP and SCS |
| A. Basu et al. 2019 [34] | HMP  20.8%  SCS  25.8% | N/A | HMP  20.8%  SCS  25.8% | HMP  2.5%  SCS  3% | - | HMP 87.1%  SCS 82.2% | HMP 96.1%  SCS 88.1% | Similar DGF in HMP and SCS despite longer CIT |
| L. Samoylova et al. 2019 [98] | HMP  25.5%  SCS  26.7% | - | - | - | HMP  9.2%  SCS  9% | - | - | HMP is associated with lower rates of rejection at 1 year on multivariable logistic regression adjusted for recipient and donor factors |
| M. Sevinc et al. 2019 [99] | 24.1% | 24.1% | - | - | - | - | - | Pulsatile and continuous machine perfusion have similar outcomes |
| M. Wszola et al. 2019 [100] | 36.6% | 36.6% | - | 2.6% | - | 90.8% | 96.8% | Shorter CIT before HMP leads to better outcomes |
| T. De Sandes Freitas et al. 2020 [58] | HMP 29.1%  SCS  55.3% | N/A | - | HMP  1%  SCS 1.9% | - |  | HMP 98.1%  SCS 96.1% | HMP after long CIT reduces DGF |
| V. Krishnamoorthy et al. 2020 [101] | HMP 23.8%  SCS  25.8% | - | - | HMP  4.9%  SCS  7.1% | - | - | - | HMP reduced the risk for DGF in kidneys with a KDPI > 30 |
| T. Kruszyna et al. 2021 [41] | HMP  21.8%  SCS  42.6% | N/A | - | HMP  3.8%  SCS  4.3% | - | HMP  93.3%  SCS  92% | - | HMP reduced DGF in CIT >12 hours |
| N. Singh et al. 2021 [102] | HMP  16%  SCS  5.1% | - | - | HMP 0.3%  SCS  0% | HMP  10.5%  SCS  2.6% | - | - | HMP parameter (flow and resistance) correlate with DGF |

**^PNF = Primary Non-function HMP = Hypothermic Machine Perfusion^**

**^DGF = Delayed Graft Function SCS = Static Cold Storage^**

**^DCD = Donation after Cardiac Death N/A = Not applicable^**

**^ECD = Extended Criteria Donor - = Not reported^**

**^DBD = Donation after Brainstem Death^**

**Assessment of publication risk for DGF in HMP vs. SCS.**


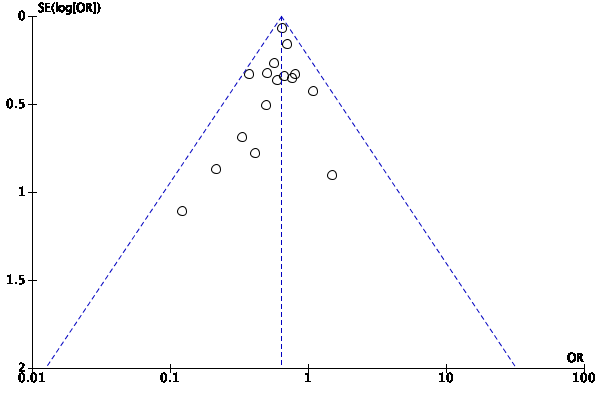


**Supplementary Figure S1.** Funnel plot for DGF in studies comparing HMP vs. SCS.

**Assessment of risk for bias for randomised controlled trials.**

**HMP+0_2_ vs. SCS and HMP vs. SCS**


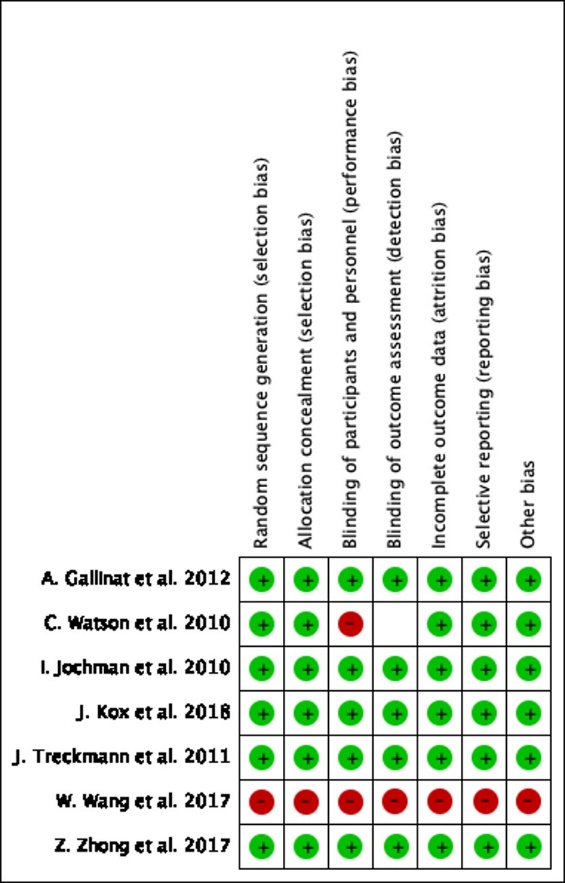


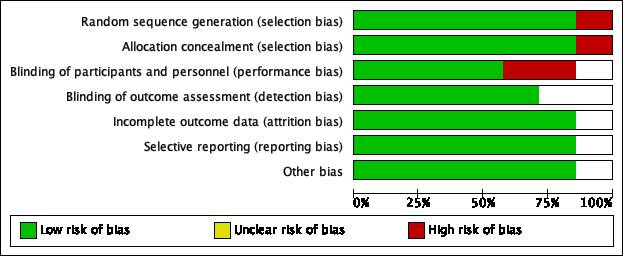


**Supplementary Figure S2.** Risk-of-bias graph and summary table. Authors' judgments about each risk-of-bias item as percentages among all included HMP vs. SCS studies.


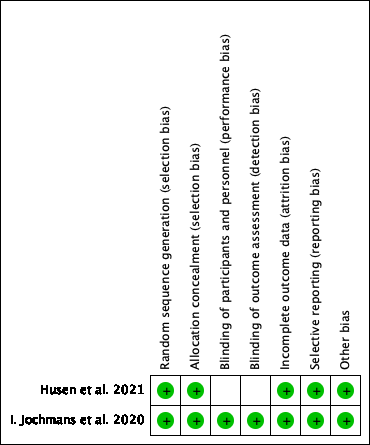

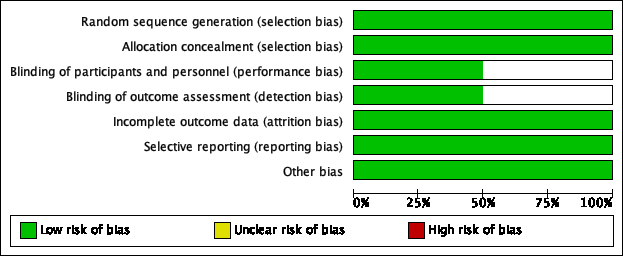


**Supplementary Figure S3.** Risk-of-bias graph and summary table. Authors' judgments about each risk-of-bias item as percentages among all included HMP+O_2_ vs. SCS studies

**Assessment of evidence level and risk for bias for comparative and non-comparative studies**

**Supplementary Table S12.** Level of evidence and quality level for bias risk (Newcastle-Ottawa score)

| HMP+O2 | | |
| --- | --- | --- |
| Study | Evidence level [104] | Newcastle-Ottawa score [105] |
| J. Houtzager et al, 2020 [78] | 4 | 5 |
| I. Jochmans et al. 2020 [12] | 1b | RCT |
| F. Meister et al. 2020 [44] | 2b | 6 |
| M. Ravaioli et al. 2020 [45] | 2b | 6 |
| Husen et al. 2021 [43] | 2b | RCT |
| NRP | | |
| O. Reznik et al. 2010 [72] | 4 | 5 |
| O. Reznik et al. 2010 [79] | 4 | 5 |
| N. Miranda-Utrera et al. 2015 [80] | 4 | 6 |
| J. Demiselle et al. 2016 [46] | 4 | 5 |
| C. Delsuc et al.2018 [19] | 4 | 6 |
| R. Pearson et al. 2021 [47] | 4 | 7 |
| HMP | | |
| F. Catena et al. 2010 [81] | 4 | 5 |
| G. Ciancio et al. 2010 [61] | 4 | 6 |
| J. Guarrera et al. 2020 [106] | 4 | 5 |
| I. Jochman et al. 2010 [28] | 1b | RCT, n.a. |
| N. Matsuno et al. 2010 [83] | 4 | 6 |
| C. Moers et al. 2010 [84] | 4 | 6 |
| C. Watson et al. 2010 [35] | 1b | RCT, n.a. |
| I. Abboud et al. 2011 [29] | 2b | 8 |
| De Vries et al. 2011 [103] | 4 | 6 |
| I. Jochmans 2011 [23] | 4 | 6 |
| J. Treckmann et al. 2011 [30] | 1b | RCT, n.a. |
| G. Ciancio et al. 2012 [85] | 4 | 6 |
| A. Gallinat et al. 2012 [31] | 1b | RCT, n.a. |
| S. Patel et al. 2012 [86] | 4 | 6 |
| R. Cannon et al. 2013 [36] | 4 | 7 |
| P. Hoogland et al. 2013 [87] | 4 | 6 |
| M. Nagelschmidt et al. 2013 [88] | 4 | 6 |
| A. Sedigh et al. 2013 [37] | 2b | 8 |
| M. Wszola et al. 2013 [89] | 1b | RCT, n.a. |
| I. Hall et al. 2014 [90] | 4 | 7 |
| M. Dion et al. 2015 [38] | 4 | 5 |
| A. Guy et al. 2015 [60] | 4 | 5 |
| A. Guy et al. 2015 [91] | 4 | 5 |
| J. Forde et al. 2016 [39] | 4 | 6 |
| S. Paloyo et al. 2016 [92] | 4 | 7 |
| C. Parikh et al. 2016 [93] | 4 | 4 |
| L. Yao et al. 2016 [32] | 4 | 5 |
| W. Wang et al. 2017 [33] | 2b | RCT, n.a. |
| Z. Zhong et al. 2017 [42] | 2b | RCT, n.a. |
| G. Chen et al. 2018 [94] | 4 | 5 |
| C. Ding et al. 2018 [95] | 4 | 5 |
| J. Kox et al. 2018 [96] | 1b | RCT, n.a. |
| C. Matos et al. 2018 [59] | 4 | 6 |
| K. Patel et al. 2018 [97] | 4 | 6 |
| M. Arlaban et al. 2019 [40] | 4 | 5 |
| A. Basu et al. 2019 [34] | 4 | 5 |
| L. Samoylova et al. 2019 [98] | 2c | 7 |
| M. Sevinc et al. 2019 [99] | 3b | 7 |
| M. Wszola et al. 2019 [100] | 3b | 7 |
| T. De Sandes Freitas et al. 2020 [58] | 3b | 7 |
| V. Krishnamoorthy et al. 2020 [101] | 4 | 6 |
| T. Kruszyna et al. 2021 [41] | 3b | 7 |
| N. Singh et al. 2021 [102] | 3b | 5 |
| NMP | | |
| M. Nicholson et al. 2013 [67] | 3b | 6 |
| P. Chandak et al. 2019 [68] | 4 | 6 |

References

1. de Vries, E.E.; Hoogland, E.R.P.; Winkens, B.; Snoeijs, M.G.; van Heurn, L.W.E. Renovascular Resistance of Machine-Perfused DCD Kidneys Is Associated with Primary Nonfunction. *Am. J. Transplant.* **2011**, *11*, 2685–2691. https://doi.org/10.1111/j.1600-6143.2011.03755.x.
2. Levels of Evidence. (March 2009). Availabe online: https://www.cebm.ox.ac.uk/resources/levels-of-evidence/oxford-centre-for-evidence-based-medicine-levels-of-evidence-march-2009 (accessed on 19 April 2023)
3. The Newcastle-Ottawa Scale (NOS) for Assessing the Quality of Nonrandomised Studies in Meta-Analyses. Availabe online: https://www.ohri.ca/programs/clinical_epidemiology/oxford.asp (accessed on 19 April 2023).
4. Garcia-Valdecasas, J.C.; Tabet, J.; Valero, R.; Taura, P.; Rull, R.; Garcia, F.; Montserrat, E.; Gonzalez, F.X.; Ordi, J.; Beltran, J.; et al. Liver conditioning after cardiac arrest: The use of normothermic recirculation in an experimental animal model. *Transpl. Int.* **1998**, *11*, 424–432. https://doi.org/10.1007/s001470050169.
